# FMK, an Inhibitor of p90RSK, Inhibits High Glucose-Induced TXNIP Expression via Regulation of ChREBP in Pancreatic β Cells

**DOI:** 10.3390/ijms20184424

**Published:** 2019-09-09

**Authors:** Jung-Hwa Han, Suji Kim, Sujin Kim, Heejung Lee, So-Young Park, Chang-Hoon Woo

**Affiliations:** 1 Department of Pharmacology, Yeungnam University College of Medicine, 170 Hyeonchung-ro, Daegu 42415, Korea; 2 Smart-Aging Convergence Research Center, Yeungnam University College of Medicine, 170 Hyeonchung-ro, Daegu 42415, Korea; 3 Department of Physiology, Yeungnam University College of Medicine, 170 Hyeonchung-ro, Daegu 42415, Korea

**Keywords:** p90RSK, FMK, TXNIP, ChREBP, INS-1

## Abstract

Hyperglycemia is the major characteristic of diabetes mellitus, and a chronically high glucose (HG) level causes β-cell glucolipotoxicity, which is characterized by lipid accumulation, impaired β-cell function, and apoptosis. TXNIP (Thioredoxin-interacting protein) is a key mediator of diabetic β-cell apoptosis and dysfunction in diabetes, and thus, its regulation represents a therapeutic target. Recent studies have reported that p90RSK is implicated in the pathogenesis of diabetic cardiomyopathy and nephropathy. In this study, we used FMK (a p90RSK inhibitor) to determine whether inhibition of p90RSK protects β-cells from chronic HG-induced TXNIP expression and to investigate the molecular mechanisms underlying the effect of FMK on its expression. In INS-1 pancreatic β-cells, HG-induced β-cell dysfunction, apoptosis, and ROS generation were significantly diminished by FMK. In contrast BI-D1870 (another p90RSK inhibitor) did not attenuate HG-induced TXNIP promoter activity or TXNIP expression. In addition, HG-induced nuclear translocation of ChREBP and its transcriptional target molecules were found to be regulated by FMK. These results demonstrate that HG-induced pancreatic β-cell dysfunction resulting in HG conditions is associated with TXNIP expression, and that FMK is responsible for HG-stimulated TXNIP gene expression by inactivating the regulation of ChREBP in pancreatic β-cells. Taken together, these findings suggest FMK may protect against HG-induced β-cell dysfunction and TXNIP expression by ChREBP regulation in pancreatic β-cells, and that FMK is a potential therapeutic reagent for the drug development of diabetes and its complications.

## 1. Introduction

The incidence of diabetes mellitus (DM) continues to increase rapidly. The disease is mainly characterized by elevated glucose levels in blood, that is, hyperglycemia. Moreover, when chronic, hyperglycemia leads to decreased insulin production, pancreatic β-cell damage, and eventually to insulin resistance [1,2]. Chronic hyperglycemia and hyperlipidemia detrimentally affect β-cell survival and function, which are defined as glucolipotoxicity [3]. Several potential mechanisms underlying glucolipotoxicity-induced β-cell dysfunction have been suggested, and these involve oxidative stress, inflammation, lipid intermediates, and endoplasmic reticulum (ER) stress [4,5,6]. 

Thioredoxin-interacting protein (TXNIP, also known as VDUP-1 for vitamin D3 up-regulated protein 1 or TBP-2 for thioredoxin binding protein 2) directly binds to the active cysteine residue of thioredoxin, and thus inhibits the expression and activity of thioredoxin [7,8]. By negatively regulating thioredoxin activity, TXNIP is involved in a wide variety of cellular processes including response to oxidative stress, cell proliferation, and apoptosis [9]. Recent reports have shown TXNIP is a critical signal that links ER stress and inflammation to metabolic diseases like cancer, heart diseases, and other metabolic diseases [9,10,11]. In particular, TXNIP is the most highly up-regulated gene in human pancreatic β-cells exposed to hyperglycemia [12]. Furthermore, TXNIP is a critical mediator of glucose-induced β-cell apoptosis, and has been reported to be overexpressed in pancreatic islets of insulin-resistant and diabetic mice [13]. In addition, deficiency of TXNIP has been found to protect against type 1 and type 2 diabetes in mice [14,15]. Taken together, TXNIP has emerged as an important factor in pancreatic β-cells, and proper regulation of its levels is necessary for β-cell survival. 

Glucose-induced TXNIP expression is mediated via carbohydrate response element-binding protein (ChREBP; a transcription factor) in pancreatic β-cells [16]. Under high glucose (HG) conditions, ChREBP is translocated to the nucleus where its binds two E-box motifs that comprise carbohydrate response element (ChoRE) on the promoters of target genes such as TXNIP, liver type pyruvate kinase (Pklr), liver pyruvate kinase (L-pk), acetyl-CoA carboxylase (ACC) and fatty acid synthase (FAS) in liver and pancreatic β-cells [17,18,19]. Several studies have reported ChREBP is regulated by post-translational modifications (phosphorylation, acetylation, and O-linked GlcNAcylation), ChREBP isoform**s** (ChREBPα and ChREBPβ), and metabolites derived from glucose [20,21,22,23,24,25,26].

p90 ribosomal S6 kinase (p90RSK) is a serine/threonine kinase member of the S6 ribosomal kinase (RSK) family, which is downstream to the extracellular signal-related kinase (ERK) signaling pathway [27]. p90RSK has two kinase domains (one at its N-terminal and the other at its C-terminal), a linker region, and an ERK docking site. Activated p90RSK interacts with numerous substrates and is involved in gene expression, protein synthesis, cell survival, proliferation, and cell cycle progression [28]. Furthermore, p90RSK can be activated by diverse stimuli, including oxidative stress and chronic hyperglycemia [29,30]. Since upregulation of p90RSK has been reported in various human diseases, aberrant activation of p90RSK plays an important role in the pathogenesis of some tumors and metabolic diseases (e.g., diabetic heart disease, atherosclerosis, diabetic nephropathy, and liver fibrosis) [31,32,33,34,35]. Thus, inhibition of p90RSK provides a promising therapeutic strategy for the diseases. Inhibitors of p90RSK like BI-D1870 and SL0101 have been developed, which target the N-terminal kinase domain (NTKD) and acts as a reversible p90RSK inhibitor [36,37], whereas FMK targets the C-terminal kinase domain (CTKD) and irreversibly inhibits p90RSK [38]. It has been reported FMK-MEA have therapeutic effects on STZ-induced diabetic mice and genetically mutated Akita mice [32]. However, the effect of p90RSK inhibitors in the pathogenesis of DM is not fully understood. In the present study, we aimed to determine whether inhibition of p90RSK by FMK might be involved in pancreatic β-cell dysfunction and TXNIP regulation, and the mechanisms involved.

## 2. Results

### 2.1. FMK Protected INS-1 Cells from High Glucose-Induced β-cell Dysfunction, Apoptosis, and Oxidative Stress

**Figure 1:**
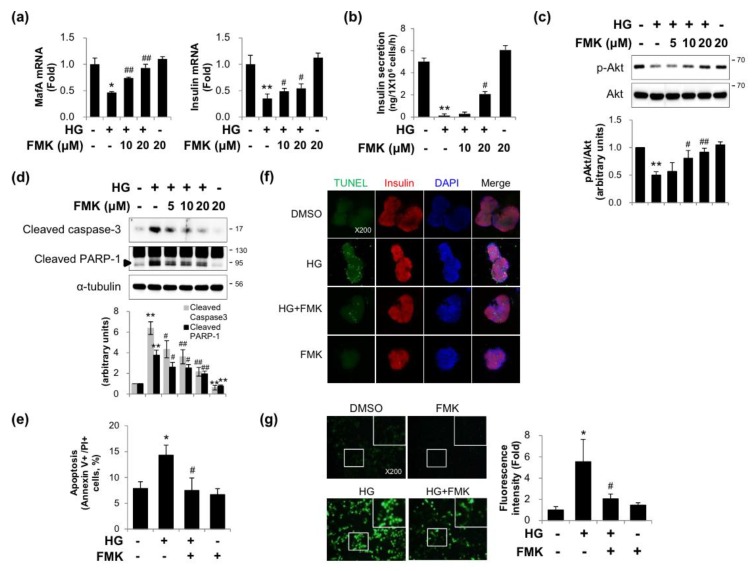
FMK pretreatment prevented high glucose-induced β-cell dysfunction, apoptosis, and oxidative stress. (**a**) INS-1 cells were pretreated with FMK (10 or 20 μM) for 1 h, and then incubated with high glucose (HG, 25 mM) for 24 h. mRNA levels of MafA and insulin were measured by qRT-PCR. Relative expression levels were normalized versus GAPDH. Results are expressed as means ± SD and are representative of three independent experiments. * *p* < 0.05 and ** *p* < 0.01 vs. non-treated controls, # *p* < 0.05 and ## *p* < 0.01 vs. HG-treated cells. (**b**) INS-1 cells were pretreated with FMK (10 or 20 μM) for 1 h, and then incubated with HG for 48 h, and then replaced with fresh medium. After 5 h recovery, the cells were subsequently simulated with KRB supplemented with HG for 1 h, and then the medium was collected for detection of glucose-stimulated insulin secretion (GSIS). Insulin secretion was determined by ELISA kit. Results are expressed as means ± SD and are representative of three independent experiments. ** *p* < 0.01 vs. non-treated controls, # *p* < 0.05 vs. HG-treated cells. (**c**,**d**) INS-1 cells were pretreated with FMK (5, 10 or 20 μM) for 1 h, and then incubated with HG for 48 h. Protein levels were measured by immunoblotting. The graph shows the densitometric quantification of western blot bands. Results are expressed as means ± SDs and are representative of three independent experiments. ** *p* < 0.01 vs. non-treated controls, # *p* < 0.05 and ## *p* < 0.01 vs. HG-treated cells. (**e**) INS-1 cells were pretreated with FMK (20 μM) for 1 h and then incubated with HG for 48 h. The status of apoptotic cell death was determined by counting cells stained with annexin V-FITC/PI using a flow cytometer. (**f**) Primary rat islets were pretreated with FMK (20 μM) for 1 h and then incubated with HG for 48 h. Cells were subjected to TUNEL staining. Representative photomicrographs showing TUNEL (apoptotic, green), insulin (pancreatic β-cells, red), and DAPI (nuclei, blue) signals and merged images (original magnification, ×200). (**g**) Representative images of ROS accumulation as determined using the fluorescent probe H2DCFDA. INS-1 cells were pretreated with FMK (20 μM) for 1 h and then incubated with HG for 48 h. These images were obtained by fluorescence microscope (original magnification, ×200). Results in bar graphs are presented as the means ± SDs of three independent experiments. * *p* < 0.05 vs. non-treated controls, # *p* < 0.05 vs. HG-treated cells.

Since p90RSK was activated in the diabetic hearts in mice, we investigated whether FMK (an inhibitor of p90RSK) could protect INS-1 cells (a pancreatic β-cell line) from HG-induced β-cell dysfunction and glucotoxicity. As shown in Figure 1a–c, insulin, MafA (a key regulator of glucose-stimulated insulin secretion) mRNA, and glucose-stimulated insulin secretion (GSIS) and *p*-Akt level decreases induced by HG were significantly recovered by FMK pretreatment. Furthermore, HG-induced increases in apoptotic marker proteins (cleaved PARP-1 and cleaved caspase-3) and Annexin V-positive/propidium iodide (PI)-positive cells were inhibited by FMK (Figure 1d,e). Consistent with the pancreatic β-cell line, HG-induced TUNEL-positive cells were reduced by FMK in primary rat islets (Figure 1f). To examine the effects of FMK on HG-induced intracellular ROS production, intracellular ROS levels were measured by H_2_DCFDA fluorescence staining. As shown in Figure 1g, FMK effectively blocked HG-induced intracellular ROS generation. These data show that FMK inhibits HG-induced β-cell dysfunction, apoptosis, and oxidative stress.

### 2.2. FMK Inhibited High Glucose-Induced TXNIP Expression in INS-1 Cells

**Figure 2:**
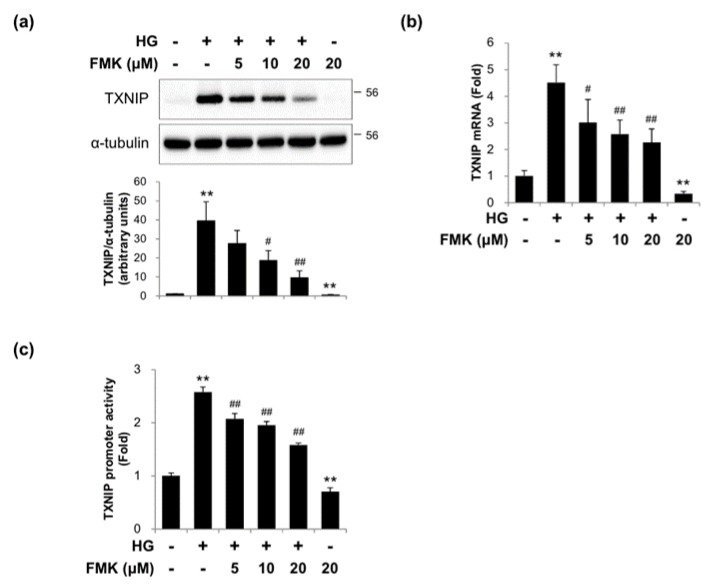
FMK inhibited high glucose-induced TXNIP expression. (**a**) INS-1 cells were pretreated with FMK (5, 10 or 20 μM) for 1 h, and then incubated with high glucose (HG, 25 mM) for 24 h. Protein levels were measured by immunoblotting using antibodies against TXNIP and α-tubulin. The graph shows the densitometric quantification of western blot bands. Results are expressed as means ± SDs and are representative of three independent experiments. ** *p* < 0.01 vs. non-treated controls, # *p* < 0.05 and ## *p* < 0.01 vs. HG-treated cells. (**b**) INS-1 cells were pretreated with FMK (5, 10 or 20 μM) for 1 h, and then incubated with HG for 24 h. mRNA levels of TXNIP were measured by qRT-PCR. Relative expression levels were normalized versus GAPDH. Results are expressed as the means ± SDs of three independent experiments. ** *p* < 0.01 vs. non-treated controls, # *p* < 0.05 and ## *p* < 0.01 vs. HG-treated controls. (**c**) INS-1 cells were transfected with a TXNIP-luc containing construct driven by full-length TXNIP promoter, and after 24 h of transfection were pretreated with FMK (5, 10 or 20 μM) for 1 h, and then incubated with HG for 24 h. Luciferase activities in cell lysates were determined using a dual luciferase reporter assay kit with a Glomax 20/20 luminometer. Transfection efficiencies were normalized versus Renilla luciferase activity derived from pRL-tk construct. Results are expressed as the means ± SDs of three independent experiments. ** *p* < 0.01 vs. non-treated controls, ## *p* < 0.01 vs. HG-treated controls.

Since TXNIP plays critical roles under diabetic conditions in vitro and *in vivo*, we examined whether FMK could inhibit TXNIP expression. HG exposure significantly increased TXNIP protein and mRNA expressions, and FMK pretreatment suppressed these HG-induced TXNIP expressions in a dose-dependent manner (Figure 2a,b). To determine the effect of FMK on TXNIP transcription, TXNIP promoter activities were assessed using a luciferase reporter assay with a luciferase reporter-containing construct driven by full-length TXNIP promoter. As shown in Figure 2c, FMK effectively inhibited the induction of TXNIP promoter activity by HG. Taken together, these results suggest FMK regulates inhibits TXNIP expression in pancreatic β cells.

### 2.3. The Actions of FMK Are Not Mediated by p90RSK, Src, or S6K1 Kinases in INS-1 Cells

**Figure 3:**
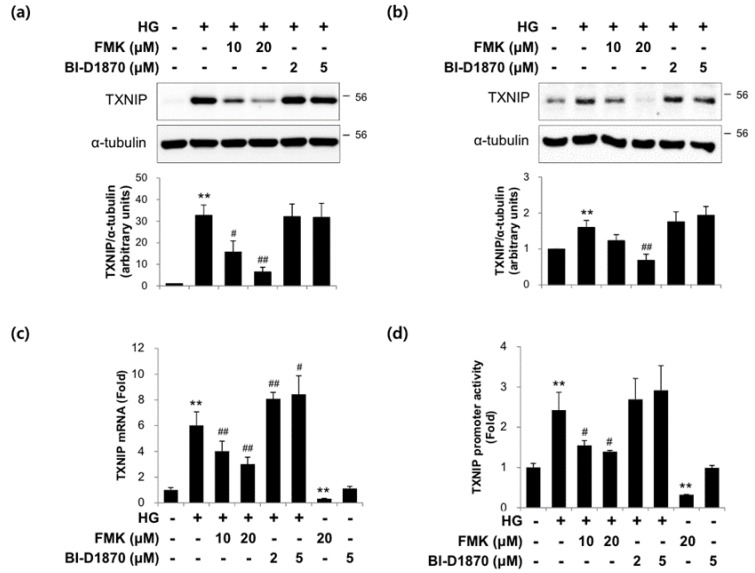
FMK (a CTKD inhibitor of p90RSK) inhibited TXNIP expression, but not BI-D1870 (a NTKD inhibitor of p90RSK). (**a**) INS-1 cells and (**b**) primary rat islets were pretreated with FMK (10 or 20 μM) or BI-D1870 (2 or 5 μM) for 1 h, and then incubated with high glucose (HG, 25 mM) for 24 h. Protein levels were measured by immunoblotting. The graph shows the densitometric quantification of western blot bands. Results are expressed as means ± SDs and are representative of three independent experiments. ** *p* < 0.01 vs. non-treated controls, # *p* < 0.05 and ## *p* < 0.01 vs. HG-treated cells. (**c**) INS-1 cells were pretreated with FMK (10 or 20 μM) or BI-D1870 (2 or 5 μM) for 1 h, and then incubated with HG for 24 h. mRNA levels of TXNIP were measured by qRT-PCR. Relative expression levels were normalized versus GAPDH. Results are expressed as the means ± SDs of three independent experiments. ** *p* < 0.01 vs. non-treated controls, # *p* < 0.05 and ## *p* < 0.01 vs. HG-treated cells. (**d**) INS-1 cells were transfected with TXNIP-luc containing construct driven by the full-length TXNIP promoter and 24 h later pretreated with FMK (10 or 20 μM) or BI-D1870 (2 or 5 μM) for 1 h, and then incubated with HG for 24 h. Luciferase activities in cell lysates were assessed using a dual luciferase reporter assay kit with a Glomax 20/20 luminometer. Transfection efficiencies were normalized versus Renilla luciferase activity derived from a pRL-tk construct. Results are expressed as the means ± SDs of three independent experiments. ** *p* < 0.01 vs. non-treated controls, # *p* < 0.05 vs. HG-treated cells.

**Figure 4:**
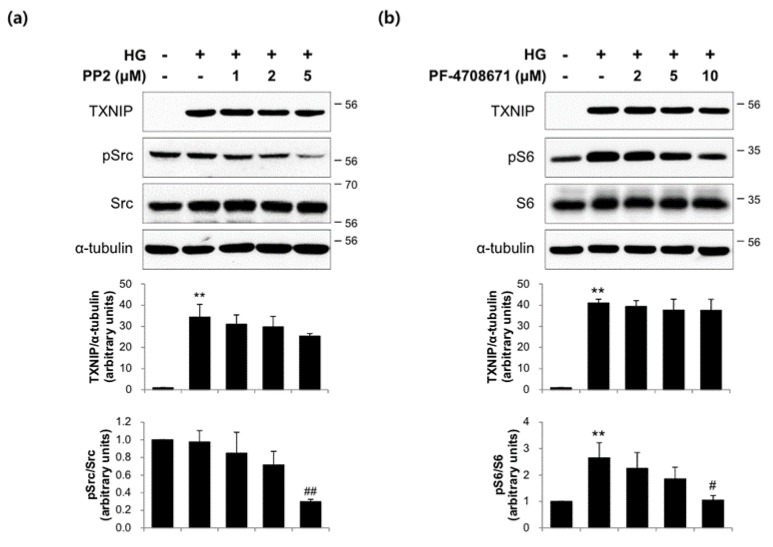
Src and S6K1 kinases were not involved on HG-induced TXNIP expression. (**a**) INS-1 cells were pretreated with PP2 (1, 2 or 5 μM; an inhibitor of Src) for 1 h, and then incubated in high glucose (HG, 25 mM) for 24 h. Protein levels were measured by immunoblotting. The graph shows the densitometric quantification of western blot bands. Results are expressed as means ± SDs and are representative of three independent experiments. * *p* < 0.05 vs. non-treated controls, # *p* < 0.05 vs. HG-treated cells (**b**) INS-1 cells were pretreated with PF-4708671 (2, 5 or 10 μM; an inhibitor of S6K1) for 1 h, and then incubated with HG for 24 h. Protein levels were measured by immunoblotting. The graph shows the densitometric quantification of western blot bands. Results are expressed as means ± SDs and are representative of three independent experiments. * *p* < 0.05 and ** *p* < 0.01 vs. non-treated controls, # *p* < 0.05 vs. HG-treated cells.

In order to confirm the role of p90RSK on TXNIP expression in response to HG, we used two pharmacological inhibitors that bind to mutually exclusive domains on p90RSK. Interestingly, unlike FMK, BI-D1870 did not suppress TXNIP protein expression in INS-1 cells (Figure 3a) and primary rat islets (Figure 3b). Also, HG-induced TXNIP mRNA and transcription were not inhibited by BI-D1870 (Figure 3c,d). These data suggest that p90RSK is not responsible for TXNIP expression by FMK. To determine whether Src or S6K1 kinases are involved in TXNIP regulation, we incubated INS-1 cells with HG in the presence or absence of PP2 (Src inhibitor) and PF-4708671 (S6K1 inhibitor), respectively. As shown in Figure 4, HG-induced TXNIP expression was not affected by PP2 or PF-4708671, indicating the effect of FMK was not mediated by Src or S6K1 kinases. 

### 2.4. FMK Inhibited the HG-Induced Nuclear Translocation and Activity of ChREBP in INS-1 Cells

**Figure 5:**
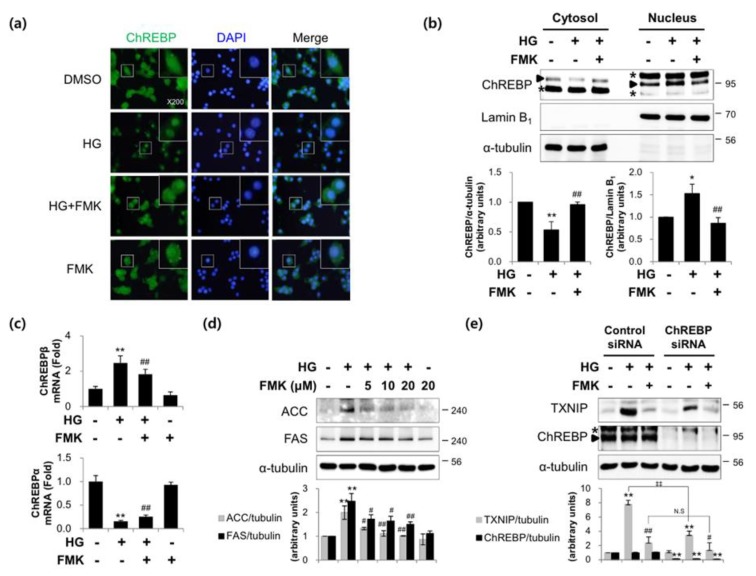
FMK reduced the HG-induced nuclear translocation and activity of ChREBP. (**a**) INS-1 cells were pretreated with FMK (20 μM) for 1 h, and then incubated with high glucose (HG, 25 mM) for 6 h. Cells were stained with anti-ChREBP antibody, and nuclei were stained with DAPI and examined under a fluorescence microscope (original magnification, ×200). (**b**) INS-1 cells were pretreated with FMK (20 μM) for 1 h and then incubated with HG for 6 h. Cytosolic and nuclear protein extracts were isolated, and protein levels were measured by immunoblotting. The asterisk indicates a non-specific band. (**c**) INS-1 cells were pretreated with FMK (20 μM) for 1 h, and then incubated with HG for 24 h. mRNA levels of ChREBPα and ChREBPβ were measured by qRT-PCR. Relative expression levels were normalized versus GAPDH. Results are expressed as the means ±SDs of three independent experiments. * *p* < 0.05 and ** *p* < 0.01 vs. non-treated controls, # *p* < 0.05 and ## *p* < 0.01 vs. HG-treated cells (**d**) INS-1 cells were pretreated with FMK (20 μM) for 1 h, and then incubated with HG for 24 h. Protein levels were assessed by immunoblotting. (**e**) INS-1 cells were transfected with control or ChREBP siRNA (100 pM). After 48 h, cells were pretreated with FMK (20 μM), and then incubated with HG for 24 h. Protein levels were measured by immunoblotting. The graph shows the densitometric quantification of western blot bands. Results are expressed as means ± SDs and are representative of three independent experiments. ** *p* < 0.01 vs. non-treated controls, # *p* < 0.05 vs. HG-treated cells, ^‡‡^*p* < 0.01; N.S., not significant. The asterisk indicates a non-specific band.

Given that the nuclear translocation of ChREBP is required for its activity, we used immunofluorescence staining and subcellular fractionation analysis to assess its translocation. As shown in Figure 5a,b, FMK pretreatment reduced the HG-stimulated nuclear accumulation of ChREBP. HG increased ChREBPβ mRNA levels and decreased ChREBPα mRNA levels and these responses were significantly impaired by FMK pretreatment (Figure 5c). In addition, HG-induced ACC and FAS protein (ChREBP target genes) level increases were inhibited by FMK pretreatment (Figure 5d). In order to prove the effects are ChREBP dependent, a knockdown of ChREBP was performed. As shown in Figure 5e, HG-induced TXNIP was reduced by knockdown of ChREBP, but FMK-mediated downregulation of HG-induced TXNIP was not further reduced by ChREBP knockdown. These results suggest FMK inhibits HG-induced TXNIP in a ChREBP-dependent manner. Taken together, these observations suggest that FMK down-regulates TXNIP by inhibiting the nuclear translocation of ChREBP.

### 2.5. Glucose 6-Phosphate is not a Target of FMK-Mediated ChREBP Regulation 

**Figure 6:**
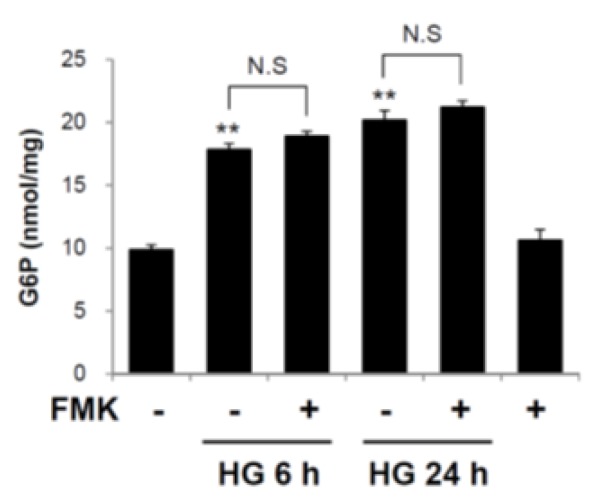
Glucose 6-phosphate was not involved in the inhibition of the HG-induced ChREBP activation by FMK. INS-1 cells were pretreated with FMK (20 μM) for 1 h, and then incubated with high glucose (HG, 25 mM) for 6 or 24 h. Intracellular concentrations of glucose 6-phosphate (G6P) were measured using a G6P colorimetric assay Kit. Results are presented as bar graphs and are the means ±SDs of three independent experiments. ** *p* < 0.05 vs. non-treated controls; N.S., not significant.

To determine whether FMK affects the formation of G6P, intracellular G6P levels were measured. As shown in Figure 6, the G6P levels were unaffected by FMK treatment, suggesting G6P formation is not involved in the inhibition of the HG-induced ChREBP activation by FMK. 

### 2.6. FMK Inhibited Tunicamycin-Induced TXNIP Expression in INS-1 Cells

**Figure 7:**
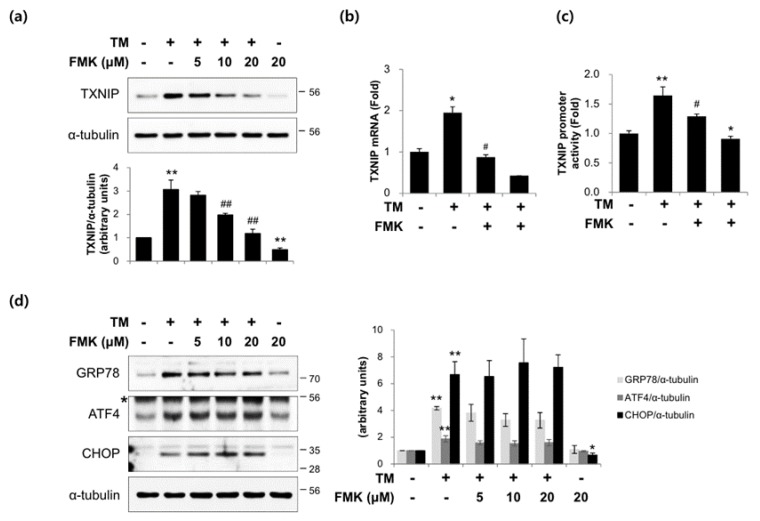
FMK pretreatment reduced tunicamycin-induced TXNIP expression. (**a**) INS-1 cells were pretreated with FMK (5, 10 or 20 μM) for 1 h, and then treated with tunicamycin (TM, 10 μM) for 9 h. Protein levels were measured by immunoblotting. The graph shows the densitometric quantification of western blot bands. Results are expressed as means ± SDs and are representative of three independent experiments. ** *p* < 0.01 vs. non-treated controls, ## *p* < 0.05 vs. HG-treated cells. (**b**) INS-1 cells were pretreated with FMK (20 μM) for 1 h, and then incubated with TM for 9 h. mRNA levels of TXNIP were measured by qRT-PCR. Relative expression levels were normalized versus GAPDH. Results are expressed as the means ± SDs of three independent experiments. * *p* < 0.05 and ** *p* < 0.01 vs. non-treated controls, # *p* < 0.05 vs. TM-treated cells. (**c**) INS-1 cells were transfected with TXNIP-luc for 24 h, pretreated with FMK (20 μM) for 1 h, and then treated with TM for 9 h. Luciferase activities in cell lysates were assessed using a dual luciferase reporter assay kit with a Glomax 20/20 luminometer. Transfection efficiencies were normalized versus Renilla luciferase activity derived from a pRL-tk construct. Results are expressed as the means ± SDs of three independent experiments. * *p* < 0.05 and ** *p* < 0.01 vs. non-treated controls, # *p* < 0.05 vs. TM-treated cells. (**d**) INS-1 cells were pretreated with FMK (5, 10 or 20 μM) for 1 h, and then treated with TM for 9 h. Protein levels were measured by immunoblotting. The graph shows the densitometric quantification of western blot bands. Results are expressed as means ± SDs and are representative of three independent experiments. * *p* < 0.05 and ** *p* < 0.01 vs. non-treated controls. The asterisk indicates a non-specific band.

Since TXNIP mediates ER stress-induced pancreatic β-cell death, we investigate whether FMK inhibits ER stress-induced TXNIP expression. INS-1 cells were exposed to tunicamycin (TM; an ER stress inducer) in the presence or absence of FMK pretreatment. TM-induced TXNIP protein and mRNA levels were inhibited by FMK (Figure 7a,b). To investigate the effect of FMK on ER stress-induced TXNIP transcription, TXNIP promoter activities were measured using a luciferase reporter assay using a luciferase reporter-containing construct driven by full-length TXNIP promoter. As shown in Figure 7c, FMK effectively inhibited the induction of TXNIP promoter activity by TM, which is also consistent with its effect on TXNIP expression. However, TM-induced levels of GRP78, ATF4, and CHOP (markers of ER stress) were unaffected by FMK (Figure 7d). Taken together, these results suggest FMK regulates TXNIP expression independently of typical ER stress response.

## 3. Discussion

In the present study, we investigated the involvement of p90RSK in high glucose (HG)-induced β-cell dysfunction and TXNIP gene expression. The major findings of the present study are that FMK (a p90RSK inhibitor) ameliorates HG-induced β-cell dysfunction and TXNIP expression independently of p90RSK by inhibiting the HG-induced nuclear translocation of ChREBP (Figure 1, Figure 2, Figure 3 and Figure 5). We also found that FMK inhibited tunicamycin (TM)-induced TXNIP expression (Figure 7). 

It has been established that chronic HG causes β-cell glucolipotoxicity, which in turn leads to lipid accumulation, impaired β-cell function, and apoptosis [5,39,40]. Furthermore, it has been reported the phosphoinositide 3-kinase/Akt (PI3K/Akt) signaling pathway plays a critical role in the regulation of β-cell mass and function [41,42], and that MafA is a key regulator of glucose-stimulated insulin secretion (GSIS) [43]. The present study showed that HG-induced reductions in insulin and MafA levels, GSIS and Akt phosphorylation and are prevented by FMK in INS-1 cells (Figure 1a–c). Taken together, these results suggest FMK might regulate insulin signaling and β-cell functions. 

It is well known that TXNIP is an endogenous inhibitor of thioredoxin, an antioxidant protein with oxidoreductase activity. TXNIP reacts with reduced thioredoxin and forms a disulfide bond between thioredoxin at Cys63 and TXNIP at Cys247 [44]. As a result, TXNIP plays a major role in redox homeostasis and promotes oxidative stress resulting in β-cell apoptosis and dysfunction. In the present study, HG-induced ROS generation was suppressed by FMK (Figure 1g). Also, HG-induced apoptosis was diminished by FMK in INS-1 cells (Figure 1d,e) and primary rat islets (Figure 1f). It has been reported that increased the ratio of Bax (an apoptosis promoter) to Bcl-2 (an apoptosis inhibitor) upregulate caspase-3 and the susceptibility of a cell to apoptosis [45,46]. Thus, it need for further study to investigate FMK might be associated with Bax/Bcl-2 ratio. Furthermore, in a recent study, it was suggested TXNIP acts as a critical signaling molecule that links oxidative stress and NLRP3 inflammasome activation [47]. These findings indicate the need for further study to determine whether FMK is involved in TXNIP-mediated inflammasome activation. Previous studies have shown TXNIP upregulation by chronic HG or TM leads to β-cell dysfunction and death via ER stress [48]. In the present study, FMK suppressed TM-induced TXNIP expression (Figure 7), but TM-induced ER stress responses were not altered by FMK, which suggests FMK might regulate TXNIP transcription downstream of ER stress. 

It has been established that p90RSK is phosphorylated by ERK1/2 at its CTKD, which results in activation of its NTKD and allows p90RSK to phosphorylate its downstream targets [27]. FMK binds to the ATP-binding site of CTKD and inhibits autophosphorylation at Ser386 of p90RSK [49]. Sapkota et al. reported that BI-D1870 specifically inhibits p90 RSK in vitro and *in vivo*, which inhibits the NTKD of p90RSK [37]. In the present study, HG-induced TXNIP expression and the transcriptional activities of TXNIP were inhibited by FMK, but not BI-D1870, which suggests p90RSK is not involved in its effect of FMK (Figure 3a,c,d). The similar results for TXNIP protein expression was obtained in primary rat islets (Figure 3b). It has also been reported that FMK inhibited other protein kinases, such as Src, Lck, Yes, Eph-A2, and S6K1 [50]. In the present study, HG-induced TXNIP expression was unaffected by PP2 or PF-4708671, which suggests the action of FMK is not mediated by Src and S6K1 (Figure 4). Therefore, further study is needed to identify the mechanism underlying the molecular target of FMK in HG-induced ChREBP activation and subsequent TXNIP induction.

Numerous studies have reported that transcription factors and TXNIP promoter sequences mediate the effects of stimuli on TXNIP gene expression. TXNIP promoter contains a ChoRE that is responsible for the glucose responsiveness of TXNIP expression [13]. Stoltzman et al. demonstrated that heterodimeric MondoA:Max-like protein X (Mlx) transcription factor shuttles from the outer mitochondrial membrane to the nucleus in response to glucose and enzymatic activity of the glycolytic pathway to directly activate TXNIP promoter through its ChoRE [51]. In particular, it has been reported that glucose-stimulated TXNIP expression is mediated by increased binding between ChREBP (a MondoA paralog) and TXNIP promoter, the recruitment of histone acetyltransferase p300, and subsequent histone H4 acetylation and the stimulation of TXNIP transcription [16]. Recent studies have shown glucose stimulation leads to an increase in ChREBPβ levels and a reciprocal decrease in ChREBPα levels in the β-cells and islets of diabetic mice [25,52]. Furthermore, ChREBP regulates gene transcription during glycolysis, gluconeogenesis, *de novo* lipogenesis, and in other processes [53,54]. As shown in Figure 5, the HG-induced nuclear translocation of ChREBP, ChREBPβ expression, and the expressions of lipogenesis marker proteins (ACC, FAS) were inhibited by FMK, suggesting FMK might regulate ChREBP transcriptional activity via upstream event of the nuclear translocation of ChREBP. In addition, HG-induced TXNIP was reduced by knockdown of ChREBP, but FMK-mediated downregulation of HG-induced TXNIP was not further reduced by ChREBP knockdown, suggesting FMK inhibits HG-induced TXNIP in a ChREBP-dependent manner (Figure 5e). Glucose-derived metabolites have also been reported to induce ChREBP transcriptional activity. In particular, G6P, xylulose 5-phosphate (X5P), and fructose 2,6-bisphosphate (F-2,6-P_2_) are all candidates for the regulation of ChREBP expression. G6P formation was found to be essential for the glucose–stimulated activation of ChREBP [24,25,55]. In the present study, G6P levels were unaffected by FMK treatment, which suggests the activation of ChREBP was regulated via events downstream of G6P formation by FMK (Figure 6). In addition, it has been reported ChREBP activation in response to glucose involves several posttranslational modifications, such as dephosphorylation of serine and threonine residues [20], O-GlcNAcylation [21], and the acetylation of lysine residues [22]. Thus, the precise mechanism underlying the regulation of ChREBP by FMK needs to be further investigated.

In summary, these results suggest FMK protects against HG-induced β-cell dysfunction and against TXNIP expression via ChREBP regulation in pancreatic β-cells, and that FMK be viewed as a potential therapeutic for the treatment of diabetes.

## 4. Materials and Methods 

### 4.1. Reagents and Antibodies 

FMK and BI-D1870 were purchased from Axon Medchem (Groningen, The Netherlands). PP2 was purchased from Cayman Chemical (Ann Arbor, MI, USA). PF-4708671 was purchased from Selleck Chemicals (Houston, TX, USA). 2′,7′-Dichlorodihydrofluorescein diacetate (H_2_DCFDA) and DAPI were purchased from Invitrogen (Carlsbad, CA, USA). TM, D-(+)-glucose and dimethyl sulfoxide (DMSO) were obtained from Sigma-Aldrich (St. Louis, MO, USA). Antibodies were purchased from the following vendors: TXNIP (MBL International, Woburn, MA, USA); ChREBP (Novus Biologicals, Centennial, CO, USA); KDEL (GRP94, GRP78) (Enzo Life Sciences, Lörrach, Germany); CREB-2 (ATF4), GADD153 (CHOP) and Src (Santa Cruz, Santa Cruz, CA, USA); Akt, p-Akt, PARP, cleaved Caspase-3, p-Src, p-S6, S6, ACC, and FAS (Cell Signaling Technology; Danvers, MA, USA); Lamin B_1_ (Invitrogen); α-tubulin (Sigma-Aldrich). 

### 4.2. Cell Culture and Islet Isolation

INS-1 cells (a rat insulinoma cell line) were cultured in RPMI 1640 (11.1 mM glucose) (Gibco, Grand Island, NY, USA) supplemented with 10% fetal bovine serum (FBS; HyClone, Logan, UT, USA), 50 U/mL penicillin, 50 μg/mL streptomycin (HyClone), and 50 μM β-mercaptoethanol (Sigma). Before treatment with high glucose (HG, 25 mM), cells were precultured under low-glucose conditions (5.5 mM) overnight. Primary islets were isolated from the pancreas of Sprague-Dawley rats by collagenase digestion as described [56], and cultured in RPMI 1640 (5.5 mM glucose) supplement with 10% FBS, 50 U/mL penicillin, 50 μg/mL streptomycin. Cells were incubated in a humidified atmosphere containing 5% CO_2_ at 37 °C.

### 4.3. Plasmid DNA Transfection and Reporter Gene Assay

TXNIP promoter-luciferase reporter (TXNIP-luc) was obtained from (Addgene plasmid #18759, Addgene, Watertown, MA, USA). For transfection, INS-1 cells were seeded into 12-well plates and incubated until 80–90% confluent. Cells were transfected in Opti-MEM (Invitrogen) with Lipofectamine 2000 (Invitrogen) mixture containing TXNIP-luc and pRL-tk constructs. Reporter activities were assayed by measuring luciferase activity using a dual luciferase kit (Promega, Madison, WI, USA) and a GloMax20/20 luminometer (Promega). Transfection efficiencies were normalized versus Renilla luciferase activities derived from pRL-tk constructs.

### 4.4. Small Interfering RNA (siRNA)

INS-1 cells were transiently transfected with 100 pM of control siRNA or siRNA against ChREBP (siChREBP) using Lipofectamine^®^ RNAiMAX (Invitrogen), according to the manufacturer’s instructions. ERK5 siRNAs were purchased from Bioneer (Daejeon, Korea). The targeting sequences of siRNA are as follows: mouse ERK5 siRNA, 5′-UACCCUUCCUGCACCCACAACCUUU-3′ [25]. Non-specific control siRNA was purchased from Bioneer and used as a negative control. Cells were harvested 48 h after siRNA transfection, and the protein expression of ChREBP were assessed by immunoblotting with specific antibodies.

### 4.5. Quantitative Real Time RT-PCR (qRT-PCR)

mRNA levels were determined by qRT-PCR. Briefly, total RNA was extracted using TRIzol^®^ reagent (Invitrogen), and reverse transcription reaction was carried out using TaqMan reverse transcription reagents (Applied Biosystems, Carlsbad, CA, USA), according to the manufacturer’s instructions. qRT-PCR was conducted using 1 μL of template cDNA, Power SYBR Green PCR Master Mix (Applied Biosystems), and an ABI PRISM 7500 unit (Applied Biosystems). Quantification was carried out using the efficiency-corrected ΔΔCq method. The primers used to amplify DNA sequences were as follows: rat TXNIP, forward 5’-ACAGAAAAGGAT TCTGTGAAGGTGAT-3´ and reverse 5´-GCCATTGGCAAGGTAAGTGTG-3´; rat MafA, forward 5´-AGGAGGTCATCCGAC TGAAACA-3´ and reverse 5´-GCGTAGCC GCGGTTCTT-3´; rat insulin, forward 5´-AGGTTGCCC GGCAGAAG-3´ and reverse 5´ -GTTGGTAGAAGGGAGCAGATGCT-3´; rat ChREBPα, forward 5′- CGACACTCACCCGCCTCTTC-3′ and reverse 5′-TTGTTCAGCCGAATCTTGTC-3′; rat ChREBPβ, forward 5′-TCTGCAGATCGCGCGGAG-3′ and reverse 5′-CTTGTCCCGGCATAGCAAC-3′; and rat GAPDH, forward 5´-TGGCACCCAGCACAATGAA-3´ and reverse 5´-CTAAGTCATAGTCCGCCT AGAAGCA-3´.

### 4.6. Western Blotting

Cells were lysed using radioimmunoprecipitation assay (RIPA) lysis buffer supplemented with 1 mM PMSF and 0.01 mM protease inhibitor cocktail (PIC). Lysates were incubated on ice for 15 min, and centrifuged at 15,000 × g for 10 min at 4°C. Protein concentrations were determined using the Bradford assay, and proteins were separated by SDS-polyacrylamide gel electrophoresis (SDS-PAGE) and transferred to polyvinylidene difluoride membranes. Membranes were immunoblotted with primary antibodies overnight at 4 °C and then immunoblotted using corresponding secondary antibodies. Protein signals were visualized using electrochemiluminescence detection reagents (Millipore, Billerica, MA, USA), according to manufacturers’ instructions.

### 4.7. Subcellular Fractionation 

Cells were lysed in lysis buffer A (10 mM HEPES, 10 mM KCl, 0.1 mM EDTA, 0.1 mM EGTA, 1 mM DTT, 1 mM PMSF) incubated on ice for 20 min, and centrifuged at 15,000 X g for 2 min at 4 °C. Supernatants were defined as cytosolic extracts. Nuclear fractions were obtained from precipitated cell pellets by adding lysis buffer B (20 mM HEPES, 0.4 M NaCl, 1 mM EDTA, 1 mM EGTA, 1 mM DTT, 1 mM PMSF) and centrifuging for 10 min. The supernatants so obtained are referred to nuclear extracts. Cellular locations of ChREBP were determined by immunoblotting with ChREBP antibody, and proper fractionation of cytosolic and nuclear compartments was confirmed using specific antibodies against tubulin and lamin B_1_, respectively.

### 4.8. Measurement of Intracellular Reactive Oxygen Species (ROS)

Intracellular ROS levels were detected using H_2_DCFDA as a fluorescence probe. INS-1 cells were seeded into 6-well plates, incubated until 80–90% confluent, pretreated with FMK for 1 h, and then stimulated with HG for 48 h. Cells were then washed with PBS, incubated for 30 min at 37°C with 10 μM H_2_DCFDA, and visualized at × 200 under a fluorescence microscope (Olympus, Tokyo, Japan). 

### 4.9. Immunofluorescence Imaging

INS-1 cells treated with HG in the presence or absence of FMK pretreatment, were fixed with 10% formalin for 10 min and permeabilized for 5 min at room temperature. Cells were then blocked with 5% normal goat serum in PBS-0.1% Tween-20, incubated with anti-ChREBP antibody (1:100) overnight at 4 °C, incubated with fluorescein isothiocyanate (FITC)-conjugated anti-rabbit IgG (Invitrogen) for 90 min, and counterstained with DAPI (Invitrogen) for 10 min at room temperature. Signals were observed using a fluorescence microscope (Olympus).

### 4.10. TUNEL Assay

Apoptosis was measured by the terminal deoxyribonucleotide transferase (TdT)-mediated dUTP nick end labeling (TUNEL) detecting in situ DNA fragmentation. TUNEL staining was performed using the in Situ Cell Death Detection Kit (Roche, Mannheim, Germany). Primary rat islets were treated with HG in the presence or absence of FMK pretreatment. Cells were washed 2 times with cold PBS, fixed with 4% paraformaldehyde for 20 min, and then permeabilized with 0.2% triton X-100 for 5 min. Cells were blocked with 5% normal goat serum in PBS-0.1% Tween-20, incubated with anti-insulin antibody (1:100) for overnight at 4 °C, and then incubated with secondary antibody labeled with Alexa Fluor 546 dye against rabbit IgG (Invitrogen, 1: 2000) for 90 min at room temperature. After washing, islets were applied to TUNEL staining following the manufacturer’s instructions. Nuclei were stained with DAPI for 30 min. Signals were observed using a confocal microscope (Leica, Buffalo Grove, IL, USA).

### 4.11. Glucose Sitmulated Insulin Secretion (GSIS)

INS-1 cells were pretreated with FMK for 1 h, and then incubated with HG for 48 h. Thereafter, the medium was carefully removed and the cells were washed with PBS, and replaced with fresh medium containing 3 mM glucose and 2% FBS. After 5 h recovery, the cells were subsequently simulated with Krebs-Ringer buffer (KRB) [119 mM NaCl, 4.75 mM KCl, 2.54 mM CaCl2, 1.2 mM MgSO4, 1.2 mM KH2PO4, 5 mM NaHCO3, and 20 mM HEPES, pH 7.4] supplemented with 25 mM glucose for 1 h, and then the medium was collected. Insulin secretion was determined by Insulin (rat) ELISA Kit (Abnova, Taipei, Taiwan), according to the manufacturer’s instructions. 

### 4.12. Flow Cytometry

INS-1 cells were seeded into 60 mm dishes and incubated until 80–90% confluent. Cells were then pretreated with FMK for 1 h, and stimulated with HG for 48 h. Cell death by apoptosis was quantified using Annexin V Apoptosis Detection Kit I (BD Bioscience, San Jose, CA, USA), according to the manufacturer’s instructions. Annexin V/propidium iodide (PI)-stained cells were promptly subjected to flow cytometry (BD FACSCanto II, BD Bioscience). Apoptotic cells were expressed as a percentage of the total number of cells.

### 4.13. Measurement of Glucose-6-Phosphate (G6P)

INS-1 cells were seeded into 60 mm dishes and incubated until 80–90% confluent. Cells were then pretreated with FMK for 1 h, and stimulated with HG for 6 or 24 h. G6P levels were measured using a glucose-6-phosphate colorimetric assay kit (Biovision, Milpitas, CA, USA). Briefly, cell homogenates were added to a 96-well plate with enzyme and substrate components and incubated for 30 min at room temperature. Measurements were obtained using a microplate reader (Bio-Rad) at 450 nm.

### 4.14. Statistical Analysis

Results in bar graphs are presented as means ± SDs. The significances of differences were determined using the Student’s *t*-test. *p* values of <0.05 were considered significantly different. All results are presented as the means of at least three independent experiments.

## Abbreviations

TXNIP	Thioredoxin-interacting protein
ChREBP	Carbohydrate response element-binding protein
HG	High glucose
TM	Tunicamycin
